# Obesity indicators as mediators of association between daytime napping and type 2 diabetes mellitus: the Guangzhou Biobank Cohort Study

**DOI:** 10.1186/s12889-021-12451-8

**Published:** 2022-01-10

**Authors:** Jing Yi Xiao, Wei Sen Zhang, Chao Qiang Jiang, Ya Li Jin, Feng Zhu, Kar Keung Cheng, Tai Hing Lam, Lin Xu

**Affiliations:** 1 School of Public Health, Sun Yat-sen University, No. 74 Zhongshan 2nd Road, Guangzhou, Guangdong Province China; 2 Guangzhou Twelfth People’s Hospital, Guangzhou, 510620 China; 3 Institute of Applied Health Research, University of Birmingham, Birmingham, UK; 4 School of Public Health, the University of Hong Kong, Hong Kong, Hong Kong

**Keywords:** Daytime napping, Obesity, Type 2 diabetes mellitus, Mediation analysis

## Abstract

**Objective:**

To examine the mediating effect of obesity indicators on the association between daytime napping and type 2 diabetes mellitus (T2DM) qualitatively and quantitatively using baseline data from the Guangzhou Biobank Cohort Study.

**Methods:**

Twenty-nine thousand three hundred fifty-five participants aged 50+ years were included in this cross-sectional study. Mediation analysis was used to assess the mediating effect of body mass index (BMI), waist circumference (WC), hip circumference (HC), waist-to-hip ratio (WHR) and waist-to-height ratio (WHtR) on the association between daytime napping and T2DM after adjustment for sex, age, education, occupation, smoking status, alcohol use and physical activity.

**Results:**

The mean (standard deviation) age of participants was 61.5 ( 7.1) years. The prevalence of T2DM and daytime napping was 12.5% and 65.2%, respectively. After adjustment for potential confounders, WC, WHR and WHtR showed partial mediating effects on the association between daytime napping and T2DM, with the proportion (95% confidence interval) of mediation effect being 10.17% (8.14–14.43%), 14.91% (11.95–21.24%) and 9.36% (7.49–13.29%), respectively. No mediating effect of BMI or HC on the association between daytime napping and T2DM was found.

**Conclusions:**

Our results showed significant mediating effects of WC, WHR and WHtR on the association between daytime napping and T2DM, suggesting that waist circumference management could be important in daytime nappers.

**Supplementary Information:**

The online version contains supplementary material available at 10.1186/s12889-021-12451-8.

## Introduction

Type 2 Diabetes Mellitus (T2DM) is a major public health concerns worldwide [1]. It was estimated that the number of patients will increase to 700 million by 2045 [2]. Of this, more than 75% will occur in developing countries [2, 3]. Daytime napping is a common habit in China and other Asian countries. A national study showed that more than one-third of Chinese adults had daytime napping [4]. Daytime napping is highly recommended and traditionally considered as a healthy lifestyle for compensating for inadequate nocturnal sleep, reducing sleepiness and improving performance and alertness [5–7]. However, the health effects related to daytime napping remain controversial [8–10]. One of our previous studies showed that frequent daytime napping was associated with a higher risk of diabetes [11], which was also supported by a number of prospective studies [4, 12–19] and Mendelian randomization [20]. However, the underlying mechanism is still unclear.

One of the possible explanations lies in obesity. Previous studies including Mendelian randomization have shown that daytime napping was likely causally associated with both general and central obesity [21–24]. Moreover, obesity has been suggested as a major causal factor of T2DM [25, 26]. Several epidemiological studies describing the association between daytime napping and diabetes have suggested a potential mediating role of obesity, but without quantifying nor examining it explicitly [4, 15, 17–19, 27]. To our knowledge, the potential mediating effect of obesity on the association between daytime napping and diabetes has not been reported in the literature. Hence, we examined the mediation effects of obesity indicators, including body mass index (BMI), waist circumference (WC), hip circumference (HC), waist-to-hip ratio (WHR) and waist-to-height ratio (WHtR), on the association between daytime napping and T2DM using data from the Guangzhou Biobank Cohort Study (GBCS).

## Methods

### Study population

The Guangzhou Biobank Cohort Study (GBCS) is a three-way collaborative prospective cohort study between the Guangzhou Twelfth People’s Hospital and the Universities of Hong Kong and Birmingham. Details of the GBCS have been described previously [28]. Briefly, all participants were recruited from a government-oriented welfare organization, the Guangzhou Health and Happiness Association for the Respectable Elders (GHHARE) from 2003 to 2008. Membership is open to permanent residents of Guangzhou aged 50 years or above for a nominal fee of 4 CNY (≈ 50 US cents) per month. Information of demographic characteristics, lifestyle, and personal medical history were collected at recruitment by face-to-face interview by trained nurses using a computer-assisted questionnaire. Anthropometric measurements were also measured by trained nurses using the standard protocol. Physical activity was categorized into inactive, minimally active, and active based on the short version of the International Physical Activity Questionnaire (IPAQ), which has been validated by us previously [29]. Fasting blood samples are obtained from all participants after overnight fasted, and glucose was determined automatically in the hospital laboratory. Ethical approval of the study was granted from the Guangzhou Medical Ethics Committee of the Chinese Medical Association, and informed consent was obtained from all participants before participation. The study was performed in accordance with the Declaration of Helsinki.

### Exposure

The exposure in this study was self-reported daytime napping, collected through a questionnaire. All participants were asked the following question: “Do you have a nap during the daytime, especially after lunch?”, if the answer is “Yes”, then further asking about the napping frequency within a week [11]. Participants were classified as non-nappers if they answered “no”, and nappers if “yes”. After accounting for daytime napping frequency, participants were further classified as non-nappers and habitual nappers (i.e., those with napping > 3 times/week) in the sensitivity analysis.

### Outcomes

The main outcome in this study was T2DM, which was defined as fasting plasma glucose ≥7.0 mmol/L, and/or self-reported diabetes diagnosed by a physician, and/or having anti-diabetic therapy [11, 30]. 3,655 (12.5%) participants had T2DM. Impaired fasting glucose (IFG) was defined as fasting plasma glucose ≥5.6 mmol/L and < 7.0 mmol/L in non-diabetic participants (*N* = 25,700) [11, 30]. Fasting plasma glucose was included as a continuous outcome variable in participants without physician-diagnosed diabetes.

### Mediators

Anthropometric measurements were performed by trained nurses using standard protocols in the morning before breakfast, including weight, standing height and WC, with light clothing and no shoes. BMI was calculated as weight (kg) divided by height squared (m^2^). WC was measured horizontally around the smallest circumference between the ribs and iliac crest, or at the navel, if no natural waistline was present. WHR was calculated by dividing waist circumference (cm) by hip circumference (cm), and the WHtR was calculated by dividing waist circumference (cm) by height (cm).

### Statistical analysis

Continuous variables were presented as means ± standard deviations (SD) or medians (25th percentile, 75th percentile). Categorical variables were presented as percentage. Pearson chi-square test and one-way analysis of variance or Kruskal-Wallis test were used to assess baseline characteristics according to daytime napping status at baseline. The multivariable linear regression was used to obtain the regression coefficients (β) and 95% confidence intervals (CI) for obesity indicators and fasting plasma glucose related to daytime napping. Potential confounders (i.e., factors associated with both daytime napping and T2DM in univariate analysis or reported in the literature) including sex, age, education, occupation, smoking status, alcohol use and physical activity, were considered in the full adjustment model. Mediation analysis was performed using the *medeff* package in Stata. Calculation of the underlying theoretical results is based on the counterfactual framework of potential outcomes [31, 32]. Briefly, the main conceptual models for the simple mediation analysis included exposure T (daytime napping), mediator M (obesity indicators), and outcome Y (T2DM). To enable comparison of the effect sizes of the different obesity indicators, each obesity indicator was transformed into Z-score before mediation analysis. All statistical analyses were 2-sided, and the *P* < 0.05 could be identified as statistical significance. Stata, version 16/MP (Stata Corp. LP, College Station, TX, USA) was used to perform all statistical processes.

## Results

Of 30,430 participants at baseline, we excluded participants with missing information on daytime napping (*N* = 306), obesity indicators (*N* = 144), diagnosis of T2DM (*N* = 218) and other potential confounders (*N* = 407), leaving 29,355 participants included in the analysis on T2DM. Moreover, for the mediation analysis involving fasting plasma glucose, we further excluded 2,337 participants with self-reported diagnosed diabetes and/or having anti-diabetic therapy to minimizing the impact of anti-diabetic therapy on fasting plasma glucose level, leaving 27,018 participants included in the analysis on fasting plasma glucose. The participants were aged from 50 to 96 years, with the mean age being 62 years.

Table 1 shows that, compared to the non-nappers, nappers had a higher proportion of men, were older, had higher education, more current smokers and current alcohol users, and were more physically active. Moreover, they also had higher levels of WC, WHR, WHtR and fasting plasma glucose than non-nappers (all *P* < 0.05). The means (SD) of fasting plasma glucose were 5.8 (1.7) mmol/L for all participants, 5.7 (1.6) mmol/L for non-nappers and 5.8 (1.7) mmol/L for nappers. No significant differences in BMI and HC were found between nappers and non-nappers. Nappers also had higher prevalence of T2DM and IFG than non-nappers (13.4 and 30.7% versus 10.7 and 27.7%, respectively, both *P* < 0.001).

**Table 1:** Demographic characteristics by daytime napping in 29,355 participants in the Guangzhou Biobank Cohort Study

Characteristics	Non-nappers	Nappers	*P*^†^
Number of participates	10,229	19,126	–
Sex, % men	19.89	31.69	< 0.001
Age, years, mean ± SD	60.7 ± 7.3	62.0 ± 7.0	< 0.001
Education, %			
Primary or below	46.17	41.29	< 0.001
Middle school	47.27	48.55	
College or above	6.56	10.16	
Occupation, %			
Manual	66.00	58.57	< 0.001
Non-manual	18.54	26.56	
Others	15.47	14.86	
Smoking status, %			
Never	84.48	78.87	< 0.001
Former	6.69	10.47	
Current	8.84	10.67	
Alcohol use, %			
Never	74.05	71.60	< 0.001
Former	3.34	3.65	
Current	22.60	24.72	
Physical activity, %			
Inactive	10.26	6.83	< 0.001
Minimally active	40.13	41.26	
Active	49.61	51.91	
Body mass index, kg/m^2^	23.8 ± 3.3	23.8 ± 3.3	0.22
Waist circumference, cm	78.1 ± 8.9	79.2 ± 9.0	< 0.001
Hip circumference, cm	90.7 ± 6.3	90.8 ± 6.4	0.21
Waist-to-hip ratio	0.86 ± 0.07	0.87 ± 0.07	< 0.001
Waist-to-height ratio	0.50 ± 0.06	0.50 ± 0.06	< 0.001
Fasting plasma glucose, mmol/l, mean ± SD (*N* = 27,018)	5.7 ± 1.6	5.8 ± 1.7	< 0.001
Type 2 diabetes mellitus, % yes	10.70	13.38	< 0.001
Impaired fasting glucose, % yes (*N* = 25,700)	27.69	30.66	< 0.001

Note: Type 2 diabetes mellitus was defined as (1) fasting plasma glucose ≥7.0 mmol/L (2) self-reported diabetes diagnosed by a physician; and/or having anti-diabetic therapy

^†^*P* value for one-way analysis of variance or chi-square test

Table 2 shows that after adjusting for all the potential confounding factors, including sex, age, education, occupation, smoking status, alcohol use and physical activity, nappers showed higher levels of WC, WHR, WHtR, and fasting plasma glucose compared with the no-nappers, with regression coefficient β (95%CI) being 0.461 (0.250–0.672) cm, 0.004 (0.003-0.006), 0.003 (0.001–0.004) and 0.073 (0.041–0.105) mmol/L, respectively. However, no association of daytime napping with BMI and HC was found. Similar associations were found for analyses by habitual napping status (Supplementary Table 1).

**Table 2:** Regression coefficients for obesity indicators and fasting plasma glucose by daytime napping status in 29,355 participants in the Guangzhou Biobank Cohort Study

Outcomes	Non-nappers	Nappers; β and 95% CI^a^
Body mass index, kg/m^2^	0.00	0.003 (− 0.077, 0.083)
Waist circumference, cm	0.00	0.461 (0.250, 0.672)^**^
Hip circumference, cm	0.00	0.074 (− 0.080, 0.229)
Waist-to-hip ratio	0.00	0.004 (0.003, 0.006)^**^
Waist-to-height ratio	0.00	0.003 (0.001, 0.004)^*^
Fasting plasma glucose, mmol/l (*N* = 27,018)	0.00	0.073 (0.041, 0.105)^**^

^a^Adjusting for sex, age, education, occupation, smoking status, alcohol use, and physical activity

^*^P < 0.01

^**^P < 0.001

Table 3 shows that the associations of daytime napping with T2DM and IFG were partly mediated by increments in WC, WHR and WHtR after similar adjustment. However, no evidence for the mediating role of BMI and HC was found. The proportions of mediation through WC, WHR and WHtR was 10.17% (95% CI, 8.14–14.43%), 14.91% (95% CI, 11.95–21.24%) and 9.36% (95% CI, 7.49–13.29%), respectively, for the association between daytime napping and T2DM; and 17.59% (95% CI, 11.23–42.69%), 19.82% (95% CI, 12.72–47.67%) and 15.81% (95% CI, 10.11–38.30%), through WC, WHR and WHtR, respectively, for the association between daytime napping and IFG.

**Table 3:** Association between daytime napping with impaired fasting glucose and type 2 diabetes mellitus with mediation by body mass index, waist circumference, hip circumference, waist-to-hip ratio, and waist-to-height ratio in 29,355 participants in the Guangzhou Biobank Cohort Study

Mediators^a^	Indirect effect (ACME) Estimate (95% CI)^b^	Direct effect (ADE) Estimate (95% CI)^b^	Total effect Estimate (95% CI)^b^	Proportion via mediation % (95% CI)^b^
**Type 2 diabetes**				
Body mass index, kg/m^2^	−0.0000 (− 0.0007, 0.0007)	0.0195 (0.0140, 0.0244)^*^	0.0195 (0.0140, 0.0208)^*^	0.04 (0.03, 0.05)
Waist circumference, cm	0.0020 (0.0011, 0.0029)^*^	0.0175 (0.0119, 0.0225)^*^	0.0195 (0.0138, 0.0244)^*^	10.17 (8.14, 14.43)^*^
Hip circumference, cm	0.0002 (−0.0002, 0.0005)	0.0194 (0.0139, 0.0243)^*^	0.0195 (0.0141, 0.0245)^*^	0.87 (0.69, 1.21)
Waist-to-hip ratio	0.0029 (0.0019, 0.0040)^*^	0.0166 (0.0110, 0.0217)^*^	0.0196 (0.0138, 0.0246)^*^	14.91 (11.95, 21.24)^*^
Waist-to-height ratio	0.0018 (0.0009, 0.0027)^*^	0.0176 (0.0120, 0.0226)^*^	0.0194 (0.0138, 0.0244)^*^	9.36 (7.49, 13.29)^*^
**Impaired fasting glucose (*N*** **= 25,700)**				
Body mass index, kg/m^2^	−0.0003 (−0.0020, 0.0012)	0.0188 (0.0084, 0.0287)^*^	0.0184 (0.0076, 0.0288)^*^	−1.82 (−4.40, −1.17)
Waist circumference, cm	0.0032 (0.0014, 0.0050)^*^	0.0151 (0.0047, 0.0252)^*^	0.0184 (0.0076, 0.0288)^*^	17.59 (11.23, 42.69)^*^
Hip circumference, cm	0.0002 (−0.0012, 0.0016)	0.0181 (0.0077, 0.0281)^*^	0.0183 (0.0076, 0.0287)^*^	1.29 (0.82, 3.11)
Waist-to-hip ratio	0.0037 (0.0022, 0.0051)^*^	0.0148 (0.0044, 0.0250)^*^	0.0185 (0.0077, 0.0289)^*^	19.82 (12.72, 47.67)^*^
Waist-to-height ratio	0.0029 (0.0011, 0.0047)^*^	0.0155 (0.0051, 0.0256)^*^	0.0184 (0.0076, 0.0288)^*^	15.81 (10.11, 38.30)^*^

Abbreviations: *ACME* average causal mediated effect, *ADE* average direct effect

^a^All mediators were standardized using Z-scores to facilitate comparison

^b^Adjusting for sex, age, education, occupation, smoking status, alcohol use, and physical activity

^*^P < 0.05

Table 4 shows the individual indirect effects of daytime napping on fasting plasma through WC (β  =  0.0106, *P*  <  0.05), WHR (β  =  0.0133, *P*  <  0.05) and WHtR (β  =  0.0095, *P*  <  0.05) individually was significant after similar adjustment. The proportion of the total effect of daytime napping mediated by WC was 14.57% (95% CI, 10.11–25.40%), by WHR was 18.22% (95%CI, 12.62–31.78%) and by WHtR was 13.12% (95% CI, 9.10–22.89%). No significant mediation through BMI and HC was found. Sensitivity analyses on non-nappers and habitual nappers showed consistent mediation results (Supplementary Table 2 and 3).

**Table 4:** Association between daytime napping and fasting plasma glucose with mediation by body mass index, waist circumference, hip circumference, waist-to-hip ratio, and waist-to-height ratio in 27,018 participants without type 2 diabetes history in the Guangzhou Biobank Cohort Study

Mediators^a^	Indirect effect (ACME) Estimate (95% CI)^b^	Direct effect (ADE) Estimate (95% CI)^b^	Total effect Estimate (95% CI)^b^	Proportion via mediation % (95% CI)^b^
Body mass index, kg/m^2^	−0.0002 (− 0.0046, 0.0039)	0.0732 (0.0431, 0.1039)^*^	0.0731 (0.0418, 0.1049)^*^	−0.24 (− 0.41, − 0.16)
Waist circumference, cm	0.0106 (0.0051, 0.0158)^*^	0.0625 (0.0324, 0.0930)^*^	0.0731 (0.0417, 0.1049)^*^	14.57 (10.11, 25.40)^*^
Hip circumference, cm	0.0016 (−0.0016, 0.0046)	0.0716 (0.0413, 0.1023)^*^	0.0731 (0.0420, 0.1048)^*^	2.13 (1.48, 3.70)
Waist-to-hip ratio	0.0133 (0.0081, 0.0182)^*^	0.0598 (0.0297, 0.0904)^*^	0.0731 (0.0417, 0.1050)^*^	18.22 (12.63, 31.78)^*^
Waist-to-height ratio	0.0095 (0.0042, 0.0146)^*^	0.0635 (0.0335, 0.0940)^*^	0.0731 (0.0417, 0.1049)^*^	13.12 (9.10, 22.89)^*^

Abbreviations: *ACME* average causal mediated effect, *ADE* average direct effect

^a^All mediators were standardized using Z-scores to facilitate comparison

^b^Adjusting for sex, age, education, occupation, smoking status, alcohol use, and physical activity

^*^P < 0.05

## Discussion

Our study showed that daytime napping was associated with T2DM and IFG through waist circumference-related obesity indicators, with the mediation effect from 9 to 20%. However, there was no evidence for the mediation through BMI or HC. Our results were generally consistent with previous studies showing napping was associated with higher risks of obesity [33] and diabetes [34] and added to the literature by quantifying the mediation effects through obesity.

In our study, waist circumference-related obesity indicators, rather than BMI nor HC, significantly mediated the association between daytime napping and T2DM/fasting plasma glucose, which has not been reported previously. A possible explanation is related to the body’s regional fat distribution, especially visceral fat accumulation. BMI is widely used as the measure of general obesity, but cannot distinguish lean and fat components of body composition. Measures of abdominal obesity (waist -related obesity indicators such as WC, WHR and WHtR) have been shown to have higher predictive ability than BMI along in predicting T2DM risk in older people [35], probably due to the adverse effects related to the excessive visceral fat accumulation [35]. Older adults may have a higher percentage of visceral fat accumulation than younger people because of the aging-related redistribution of fat mass to a more central deposition [36, 37], and become more vulnerable to T2DM [38].

The present study also supported the positive association between daytime napping and obesity (especially abdominal obesity) [33, 39, 40]. Compared with non-napping, daytime napping was associated with a greater risk of obesity in the Hispanic Community Health Study/Study of Latinos Sueño Ancillar Study [33]. The Study of Osteoporotic Fractures also showed a dose-response association between a longer duration of daytime napping and a higher risk of obesity [39], which was also supported by the China Health and Retirement Longitudinal Study [40].

Some possible biological mechanisms have been proposed for the positive association of daytime napping with obesity and further development of T2DM. First, napping could increase adiposity through circadian rhythm-related neuroendocrine dysfunction and behavior-related changes [41, 42], which may further lead to a higher risk of T2DM [43]. Moreover, the sympathetic nervous system might be activated after daytime napping and subsequently increase the cortisol level [44], which may promote eating behavior and fat deposition and lead to weight gain [45]. The fat deposition tended to occur in the abdominal area through fat redistribution from peripheral to central depots [46]. In addition, daytime napping is directly associated with a longer duration bedtime, which may also decrease energy expenditure and cause fat deposition [47]. Furthermore, higher level of inflammatory biomarkers may have also played a role in the association of daytime napping with obesity and T2DM [48–50].

There were some limitations in this study. First, the causal pathway from napping to obesity and then to diabetes could not be confirmed in this study. However, previous prospective cohort studies and Mendelian randomization have reported consistently positive associations of daytime napping with T2DM [4, 12–19, 24] and obesity [21–24, 27]. These results support the causal link being inferred. Moreover, of these studies mentioned above, some proposed the possible mediation via obesity but without examining the mediation effects explicitly [4, 15, 17–19, 27]. Our study adds to the previous literature by investigating and quantifying the mediation role of napping. Moreover, reverse causation (i.e., patients with diabetes tended to have daytime napping) cannot be ruled out, although daytime napping is a long-accepted culture in China, which might have been formed since young age. Notably, previous prospective cohort studies and Mendelian randomization have shown a positive association from daytime napping to obesity, but not vice versa [21–24], supporting the causal direction from napping to obesity. Second, assessment of daytime napping was self-reported and thus misclassification error was possible. Further studies using more accurate and detailed assessment of napping are needed. Third, as participants of this study were volunteers of older people in southern China, sample representativeness might also be a concern. However, within age-group, our participants had similar prevalence of T2DM and IFG to the nationally representative samples of Chinese [51]. Finally, residual confounding cannot be completely ruled out, although we have adjusted for many potential confounders reported in the literature to minimize the confounding effect.

## Conclusions

Our study firstly identified and quantified the mediating role of increased WC, WHR and WHtR in the association of daytime napping with T2DM. The findings provided new insights in understanding the possible mechanisms involved in the association between daytime napping and T2DM.

## Supplementary Information

**Additional file 1: Supplementary Table 1.** Regression coefficients for obesity indicators and fasting plasma glucose by daytime napping status in 29,355 participants in the Guangzhou Biobank Cohort Study. **Supplementary Table 2.** Association between daytime napping with impaired fasting glucose and type 2 diabetes mellitus with mediation by body mass index, waist circumference, hip circumference, waist-to-hip ratio and waist-to-height ratio in 29,355 participants in the Guangzhou Biobank Cohort Study. **Supplementary Table 3.** Association between daytime napping and fasting plasma glucose with mediation by body mass index, waist circumference, hip circumference, waist-to-hip ratio and waist-to-height ratio in 27,018 participants without type 2 diabetes history in the Guangzhou Biobank Cohort Study.

## Data Availability

Due to privacy or ethical restrictions, the data that support the findings will be made available on requests from the Guangzhou Biobank Cohort Study Data Access Committee (kh.ukh@atadscbg). The datasets used and/or analysed during the current study are available from the corresponding author on reasonable request.
