# Supplementary material for: Obesity indicators as mediators of association between daytime napping and type 2 diabetes mellitus: the Guangzhou Biobank Cohort Study
Source: BMC Public Health. 2022 Jan 10;22:56. doi: 10.1186/s12889-021-12451-8 (PMC8744231; doi:10.1186/s12889-021-12451-8)
Supplement: Supplementary file 1 — Additional file 1: Supplementary Table 1. Regression coefficients for obesity indicators and fasting plasma glucose by daytime napping status in 29,355 participants in the Guangzhou Biobank Cohort Study. Supplementary Table 2. Association between daytime napping with impaired fasting glucose and type 2 diabetes mellitus with mediation by body mass index, waist circumference, hip circumference, waist-to-hip ratio and waist-to-height ratio in 29,355 participants in the Guangzhou Biobank Cohort Study. Supplementary Table 3. Association between daytime napping and fasting plasma glucose with mediation by body mass index, waist circumference, hip circumference, waist-to-hip ratio and waist-to-height ratio in 27,018 participants without type 2 diabetes history in the Guangzhou Biobank Cohort Study. [file 12889_2021_12451_MOESM1_ESM.docx]

Supplementary Table 1. Regression coefficients for obesity indicators and fasting plasma glucose by daytime napping status in 29,355 participants in the Guangzhou Biobank Cohort Study.

| Outcome | Non-nappers | Habitual nappers; β and 95% CI^†^ |
| --- | --- | --- |
| Body mass index, kg/m^2^ | 0.00 | -0.000 (-0.077, 0.076) |
| Waist circumference, cm | 0.00 | 0.416 (0.214, 0.619)^**^ |
| Hip circumference, cm | 0.00 | 0.071 (-0.077, 0.219) |
| Waist-to-hip ratio | 0.00 | 0.004 (0.003, 0.005)^**^ |
| Waist-to-height ratio | 0.00 | 0.002 (0.001, 0.004)^*^ |
| Fasting plasma glucose, mmol/l (N=27,018) | 0.00 | 0.070 (0.040, 0.101)^**^ |

^†^: Adjusting for sex, age, education, occupation, smoking status, alcohol use and physical activity.

^*^P<0.01, ^**^P<0.001.

Supplementary Table 2. Association between daytime napping with impaired fasting glucose and type 2 diabetes mellitus with mediation by body mass index, waist circumference, hip circumference, waist-to-hip ratio and waist-to-height ratio in 29,355 participants in the Guangzhou Biobank Cohort Study.

| Mediators ^†^ | Indirect effect (ACME) Estimate (95% CI) ^‡^ | Direct effect (ADE) Estimate (95% CI) ^‡^ | Total effect Estimate (95% CI) ^‡^ | Proportion via mediation % (95% CI) ^‡^ |
| --- | --- | --- | --- | --- |
| **Type 2 diabetes** | | | | |
| Body mass index, kg/m^2^ | -0.0000 (-0.0008, 0.0007) | 0.0248 (0.0176, 0.0321)^*^ | 0.0248 (0.0174, 0.0323)^*^ | -0.11 (-0.16, -0.08) |
| Waist circumference, cm | 0.0022 (0.0011, 0.0032)^*^ | 0.0225 (0.0153, 0.0297)^*^ | 0.0246 (0.0171, 0.0321)^*^ | 8.81 (6.76, 12.70)^*^ |
| Hip circumference, cm | 0.0002 (-0.0002, 0.0006) | 0.0246 (0.0173, 0.0319)^*^ | 0.0248 (0.0175, 0.0322)^*^ | 0.80 (0.62, 1.13) |
| Waist-to-hip ratio | 0.0032 (0.0020, 0.0044)^*^ | 0.0215 (0.0143, 0.0287)^*^ | 0.0247 (0.0171, 0.0322)^*^ | 12.99 (9.97, 18.80)^*^ |
| Waist-to-height ratio | 0.0018 (0.0007, 0.0029)^*^ | 0.0228 (0.0156, 0.0301)^*^ | 0.0246 (0.0171, 0.0321)^*^ | 7.39 (5.66, 10.64)^*^ |
| **Impaired fasting glucose (N=25,700)** | | | | |
| Body mass index, kg/m^2^ | 0.0001 (-0.0016, 0.0017) | 0.0203 (0.0097, 0.0311)^*^ | 0.0204 (0.0093, 0.0316)^*^ | 0.53 (0.34, 1.16) |
| Waist circumference, cm | 0.0034 (0.0015, 0.0052)^*^ | 0.0170 (0.0064, 0.0278)^*^ | 0.0204 ( 0.0094, 0.0315)^*^ | 16.63 (10.71, 36.16)^*^ |
| Hip circumference, cm | 0.0006 (-0.0008, 0.0020) | 0.0197 (0.0090, 0.0305)^*^ | 0.0204 (0.0094, 0.0315)^*^ | 3.20 (2.06, 6.92) |
| Waist-to-hip ratio | 0.0035 (0.0020, 0.0049)^*^ | 0.0170 (0.0063, 0.0278)^*^ | 0.0205 (0.0094, 0.0316)^*^ | 17.08 (11.04, 37.05)^*^ |
| Waist-to-height ratio | 0.0027 (0.0009, 0.0045)^*^ | 0.0176 (0.0070, 0.0283)^*^ | 0.0203 (0.0093, 0.0314)^*^ | 13.48 (8.68, 29.43)^*^ |

^†^: All mediators were standardized using Z-scores to facilitate comparison.

^‡^: Adjusting for sex, age, education, occupation, smoking status, alcohol use, and physical activity.

Abbreviations: ACME, average causal mediated effect; ADE, average direct effect.

^*^P<0.05.

Supplementary Table 3. Association between daytime napping and fasting plasma glucose with mediation by body mass index, waist circumference, hip circumference, waist-to-hip ratio and waist-to-height ratio in 27,018 participants without type 2 diabetes history in the Guangzhou Biobank Cohort Study.

| Mediators ^†^ | Indirect effect (ACME) Estimate (95% CI) ^‡^ | Direct effect (ADE) Estimate (95% CI) ^‡^ | Total effect Estimate (95% CI) ^‡^ | Proportion via mediation % (95% CI) ^‡^ |
| --- | --- | --- | --- | --- |
| Body mass index, kg/m^2^ | 0.0005 (-0.0037, 0.0045) | 0.0697 (0.0407, 0.0992)^*^ | 0.0702 (0.0402, 0.1008)^*^ | 0.74 (0.51, 1.29) |
| Waist circumference, cm | 0.0104 (0.0051, 0.0153)^*^ | 0.0598 (0.0310, 0.0892)^*^ | 0.0702 (0.0401, 0.1008)^*^ | 14.81 (10.28, 25.84)^*^ |
| Hip circumference, cm | 0.0019 (-0.0011, 0.0049) | 0.0683 (0.0392, 0.0979)^*^ | 0.0702 (0.0403, 0.1007)^*^ | 2.75 (1.91, 4.78) |
| Waist-to-hip ratio | 0.0125 (0.0075, 0.0172)^*^ | 0.0577 (0.0288, 0.0871)^*^ | 0.0702 (0.0400, 0.1009)^*^ | 17.83 (12.35, 31.12)^*^ |
| Waist-to-height ratio | 0.0085 (0.0033, 0.0133)^*^ | 0.0617 (0.0328, 0.0911)^*^ | 0.0702 (0.0400, 0.1007)^*^ | 12.10 (8.40, 21.12)^*^ |

^†^: All mediators were standardized using Z-scores to facilitate comparison.

^‡^: Adjusting for sex, age, education, occupation, smoking status, alcohol use, and physical activity.

Abbreviations: ACME, average causal mediated effect; ADE, average direct effect.

^*^P<0.05.
